# A comprehensive review on nutritional interventions and nutritive elements: Strengthening immunity for effective defense mechanism during pandemic

**DOI:** 10.1002/fsn3.4138

**Published:** 2024-05-03

**Authors:** Sonal Prasad, Vinay Kumar Pandey, Kunal Singh, Rafeeya Shams, Rahul Singh, Gulden Goksen

**Affiliations:** ^1^ Faculty of Bio‐Sciences IBST, SRMU Lucknow India; ^2^ RDC, Biotechnology Department Manav Rachna International Institute of Research and Studies (Deemed to Be University) Faridabad India; ^3^ Institute of Bio Science and Technology Shri Ramswaroop Memorial University Lucknow India; ^4^ Department of Food Technology and Nutrition Lovely Professional University Phagwara India; ^5^ Department of Bioengineering Integral University Lucknow India; ^6^ Department of Food Technology, Vocational School of Technical Sciences at Mersin Tarsus Organized Industrial Zone Tarsus University Mersin Turkey

**Keywords:** immunity, minerals, polyphenols, viral infection, vitamins

## Abstract

The pandemic has brought attention to the importance of a healthy immune system in preventing infectious diseases. In this in‐depth review, the process by which nutritional interventions and fundamental nutrients affect immune function has been discussed with the goal of enhancing the body's natural defenses against viral infections. We explored the complex interplay between diet and immunology, highlighting the essential nutrients, vitamins, minerals, and bioactive substances that are crucial for enhancing immune response. We also investigated the effect of dietary patterns and supplementation methods on immune function. We assessed the effectiveness and potential mechanisms of action of various nutritional therapies in modifying immune responses through a thorough examination of scientific literature. Additionally, we go through the significance of individualized nutrition and highlight possible factors to consider for vulnerable groups, such as the elderly and people with chronic conditions. This review attempts to provide a thorough understanding of the role of diet in boosting immunity by synthesizing available research. It also offers insights into practical methods for enhancing the immune function during the current epidemic and in the future.

## Graphical abstract

The phase of pandemic has brought attention to how important a healthy immune system is in preventing infectious diseases. In this in‐depth review, the process that how nutritional interventions and fundamental nutrients affect immune function has been mentioned with the goal of enhancing the body's natural defenses against viral infections.

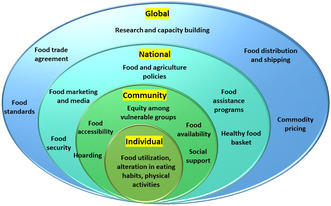

## 1 INTRODUCTION

**FIGURE 1:**
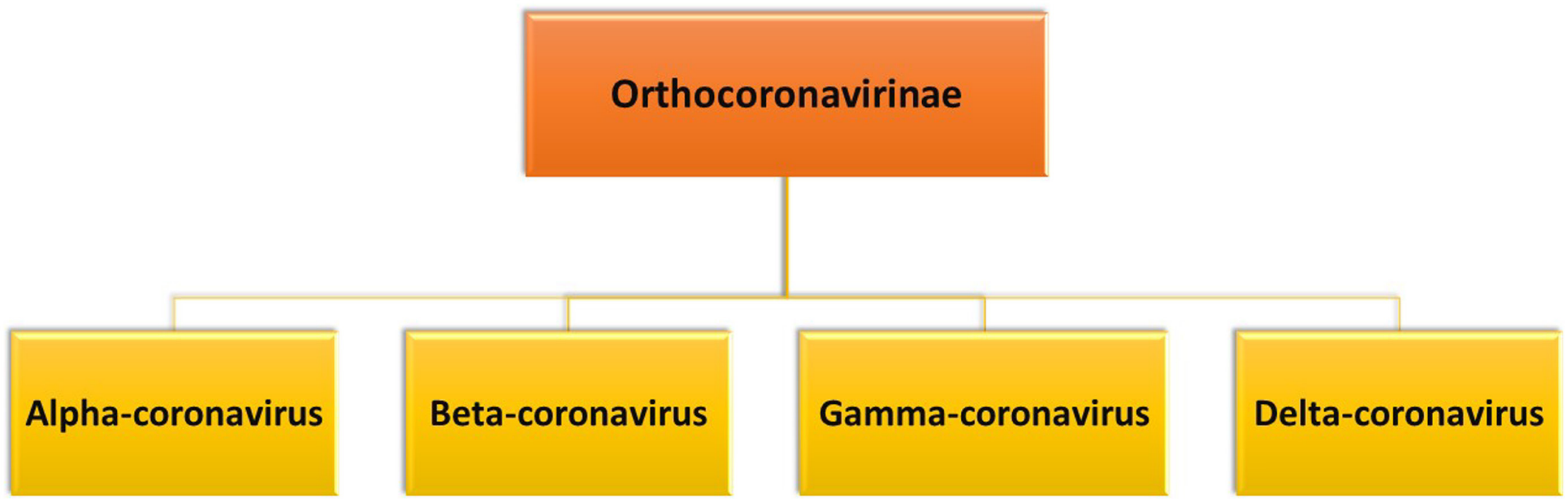
Classification of corona viruses.

Infectious disease had caused a lot of death in the pasts, more than 100 year ago, the “Spanis” influenza viruses of 1918–1919 was the foremost disastrous breakout within the recorded history The whole world got affected from this and around 20 million people died from this infectious disease. At the end of 2019, a new flu‐like virus emerged in China, commonly known as COVID‐19, which is associated with the SARS and Middle East respiratory syndrome (MERS) viruses (Cohen & Normile, 2020; Zhu et al., 2020), and transmission from humans to humans has become evident in cases where people come in close contact, especially in travel‐related cases in many countries and regions outside the People's Republic of China, including France, Germany, Thailand, Asian nations, Japan, Canada, Vietnam, and the US, such as ports and Taiwan (Ralph et al., 2019). The WHO named this new virus, which emerged in 2019, the 2019 novel viral virus, on January 12, 2020. The prevalence of COVID‐19 was reported to be approximately 82%, similar to that of SARS. COVID19 became a pandemic as it spreads worldwide, leading to numerous deaths affecting mankind (Chen et al., 2020; Zhang et al., 2020). The viral family (*Coronaviridae*) is the largest in the order *Nidovirales* consisting of two subdivisions: *Orhtocoronavirinae* and *Letovirinae. Orthocoronavirinae* is divided into four subfamilies, namely *betacoronavirus, alphacoronavirus*, *deltacoronavirus*, and *gammacoronavirus* (Figure 1). Alphacoronaviruses and betacoronaviruses are predominantly present in mammals, whereas *gammacoronaviruses* and *deltacoronaviruses* have originated mainly in birds. Earlier, it was considered responsible for enzootic infections that were found to be present in an animal community, but now it has been observed in human beings as well in recent years. In 2002, SARS's resurgence and MERS's emergence in 2012 both contributed to a rise in mortality rates by devastating people and killing large numbers of people practically everywhere in the world (Schoeman & Fielding, 2019). According to previous studies, bats serve as natural reservoirs of viruses. SARS and MERS are elements of the β‐coronavirus family (Zumla et al., 2015). The genome of the virus could be a single‐stranded positive‐form RNA whose sequence analysis proved that it is the distinctive genome structure of viruses that possess the β‐corona viruses, including SARS COVID and MERS COVID (Chen et al., 2020).

To date, no specific medicines or vaccines have been available to combat COVID‐19 and prevent the detrimental effects of this pandemic. Therefore, in the dearth of medication and treatment, it has become very important to protect ourselves by strengthening immunity by focusing on vitamins, minerals, probiotics, functional foods, and Ayurvedic herbs. Vitamin and mineral deficiencies have been shown to play a role in various diseases. This study aimed to analyze the role of various food components in the enhancement of immunity.

## 2 PANDEMIC OF VIRUSES

Viruses ‘widespread in 1918–1919 were a major disastrous break in the record of history. The characteristics of the 1918 global influenza A strain were identified in reference to its unusual virulence with the investigation of the complete sequence of the hemagglutinin (HA) gene of 1918 viruses and determination of the whole coding sequence of the neuraminidase gene of the 1918 virus. These extremely recombinant viruses, which express the 1918 viral hem agglutinin, have the potential to infect all respiratory systems, particularly the lungs, and induce the maximum quantity of macrophage‐obtained cytokines and chemokines, leading to the intrusion of inflammatory cells and severe hallmarks and hemorrhage of the sickness cause during the initial widespread infection.

As reported in a previous study, the 1918 pandemic virus was ready to replicate in the absence of trypsin, causing the death of mice and embrocated chicken eggs exhibiting a fast‐growing phenotype in cells of bronchial epithelial masses. In the absence of a particular treatment and drug, the hem agglutinin (HA), matrix (M), and neuraminidase (NA) genes of the 1918 viruses were reconstructed, and the alteration of influenza virus bearing the 1918 NA, HA, or M series was generated; it was observed that the re‐joined viruses possessing both the 1918 NA and 1918 HA were virulent, as reported in in vivo studies of mice, proving that the antiviral plan adopted would be active in restraining the hazards of a re‐emergent 1918 or 1918‐like virus. Several countries worldwide are facing the problem of COVID19 and many of them have experienced lockdowns. The spread through people came into close contact, especially in travel‐related cases in multiple countries such as China, France, Germany, Japan, Thailand, Canada, the United States, Vietnam, and South Korea, as well as Hong Kong and Taiwan (Ralph et al., 2019).

## 3 SIGNIFICANCE OF NUTRITIONAL ELEMENTS AND COMPOUNDS AGAINST VIRAL INFECTION

**FIGURE 2:**
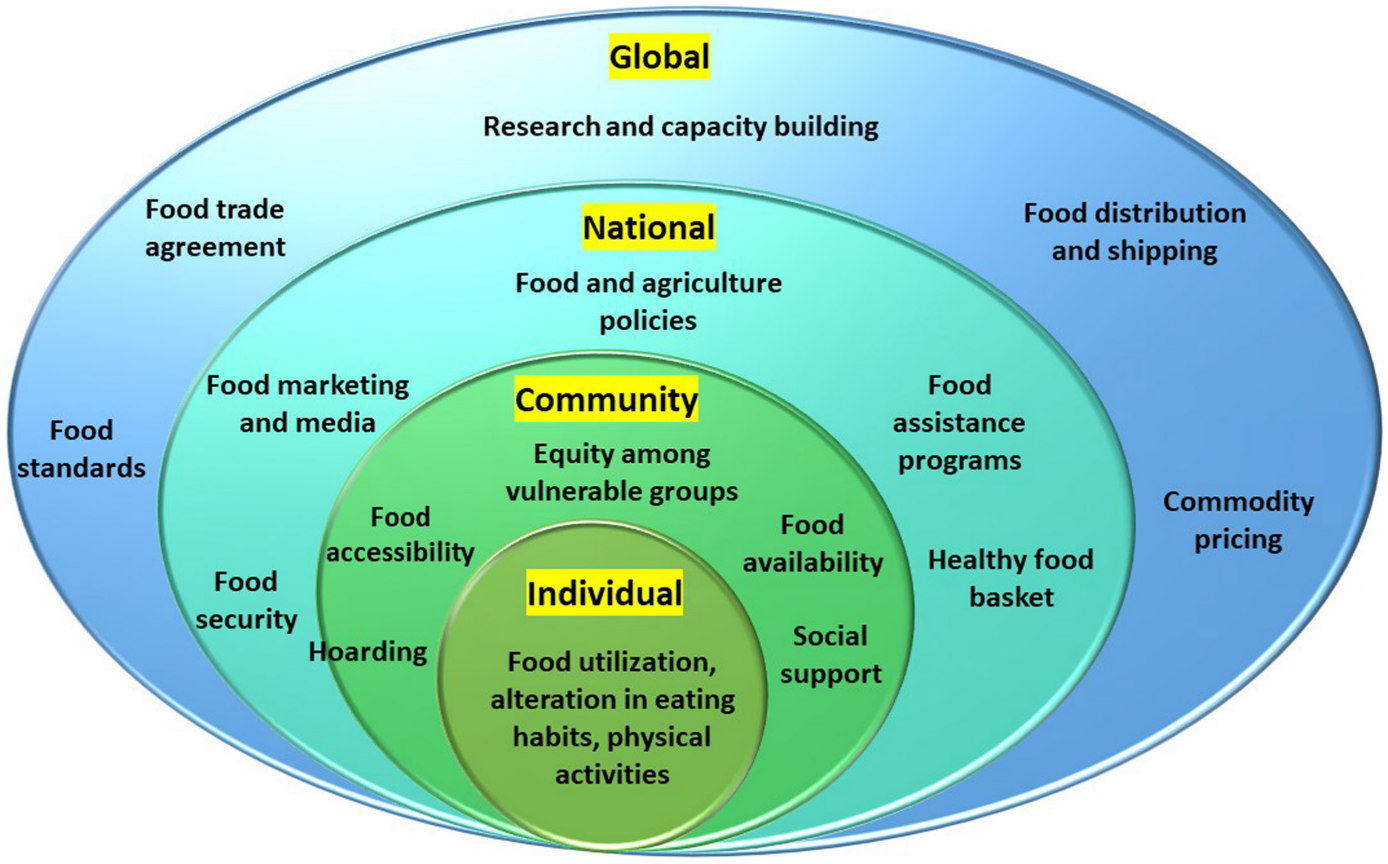
A multilevel framework that makes use of the individual, community, national, and international levels of the ecological health model to assist with food and nutrition security during the viral pandemic.

Currently, various natural agents are being used against diseases including COVID19. Maintenance of optimal nutrition is important for the enhancement of physical and mental health during COVID19 at single, group, national, and universal levels (Figure 2).

### 3.1 Roles of vitamin A

Vitamin A is one of the most potent nutrient among the fat‐soluble vitamins required for normal immune function and regulation. It belongs to the retinoid subgroup, comprising the biotic pursuit of whole‐trans‐retinol. Preformed fat‐soluble vitamins are found mostly in animal sources, such as eggs, liver, dairy products, and fish liver oils as retinal palpitates, whereas carotenoids that are converted into retinols are predominantly found in deep‐orange fruits and vegetable foodstuffs (vegetables such as dark‐green leafy). Vitamin A is extremely important in boosting immunity. During fat‐soluble vitamin A Insufficiency, the reduction in the number of goblet cells in epithelial tissues results in a decline in mucous secretions with their antimicrobial components. During Vitamin A deficiency (VAD), cells lining safe tissue areas do not renew and transform because they accumulate keratin and flatten. Both factors, the reduction in mucous diminishing and secretions of the cellular probity, affect the body's abilities in a detrimental manner by resisting the intrusion from potentially pathogenic organisms, including viruses and bacteria. Pathogens weaken the system by disrupting the assembly of various protective secretions and cells. Vitamin A enhances immunity and plays a key role in humoral and cellular immune processes, including the formation of antibodies and the function of natural killer cells, neutrophils, monocytes or macrophages, B lymphocytes, and T lymphocytes, thereby protecting the body against severe bacterial and viral infections (Huang et al., 2018). Clinical analyses have proven that Vitamin A effectively decreases mortality in communicable diseases, such as diarrheal disease, measles, measles‐related pneumonia, malaria, and HIV infection. As the immune effect differs in various infections, axerophthol greatly modulates immune function through the expression of keratin and mucin, lymphopoiesis, cytokine production, apoptosis, functions of neutrophils, NK cells, macrophages or monocytes, B lymphocytes and T lymphocytes, and production of immunoglobulin. Research has proven that vitamin A increases specific IgG antibody levels and the total series of lymphocytes, reducing despair in measles. The increased consumption of vitamin A is currently recognized as a vital tool for the treatment of acute measles viral infections, decreasing both mortality and morbidity. One study showed that ophthalmic factor deficiency in HIV‐positive pregnant women is similar to the transmission of HIV infection from mothers to children, and low levels of retinol in plasma may simply indicate the presence of consequential disastrous infection, which may lead to increased transmission from HIV‐infected mothers to children. In another study, retinoic acid (RA), a derivative considered to be a subsidiary for the induction of cytotoxic‐derived T cells; therefore, the potent complementary effects that RA has shown on the induction of cell‐mediated cytotoxicity at less amount is also the rationale for its anticarcinogenic activity (Oliveira et al., 2018). An in vivo study on rats has implicated that retinol graph is a necessary factor for humeral action to certain forms of antigen and recommends that antibody preparation to T‐cell‐dependent antigens and capsular polysaccharides depends on adequate retinol status (Pasatiempo et al., 1990).

It is well known that the enzymes alcohol dehydrogenate and aldehyde dehydrogenase play a key role in the conversion of retinol to retinoic acid, which consumes alcohol regularly beyond the traditional needs; the amount of ethanol often increases beyond the amount of alcohol dehydrogenases being produced in the hepatic cells, causing reduced retinoic acid production, resulting in antiophthalmic factor needs in liver cells. During viral hepatitis virus (HCV) fight, host cells begin building interferons, which are considered to be natural viral replication inhibitors that also result in the activation of the interferon genes of the nearby cells, also referred to as interferon‐stimulated genes (Dustin et al., 2016). An in vitro study has proven that Vitamin A remarkably reduce measles‐associated morbidity and mortality by replicating MeV through a RARα‐ and sort IFN‐dependent mechanism. RIG‐I expression, which is activated by measles virus RNA, is induced by retinoids that are important for IFN signaling. Retinoid signaling together with IFN significantly induces high levels of RIG‐I, as RIG‐I is necessary for retinoid‐MeV antiviral results. Hence, based on extensive research on the immune‐boosting effects of Vitamin A ability to prevent viruses, we can conclude that Vitamin A effective in the treatment of this emerging virus by acting as a safeguard against the harmful effects of viruses and preventing the body from its ill‐effects (Dorokhov et al., 2015).

### 3.2 Vitamin B complex in immunity boost up

The vitamin B complex consists of a gaggle of vitamins that do not seem to be stored within the body and are eliminated within the urine, which are collectively referred to as water‐soluble vitamins. Thiamine, Riboflavin, pyridoxine, biotin, niacin, cyanocobalamin, and folate are the most powerful B‐complex vitamins known for their rich antioxidant properties (Roje, 2007). Research has proven that the addition of either vit‐B complex or pteroylmonoglutamic acid was shown to be highly effective when employed in combination instead of when used singly and had lower potency than their combination. Supplementation of both B‐complex and B‐complex vitamins can decrease the detrimental effects of ribavirin (PIFN/RBV) and PEGylated interferon α‐2a therapy in patients with chronic hepatitis C, improving health‐related quality of life. PIFN/RBV has been employed to treat hepatitis C virus (HCV), but exhibits some blood‐related disorders due to the combination of B‐complex vitamins and folic acids (Ashoush et al., 2015). Cyanocobalamin supplementation notably improved sustained viral response rates in HCV‐infected patients in the context of antiviral therapy. Large doses of vitamin B supplementation (50 or 100 mg/day) could restore the loss of responsiveness of the physiologically active coenzyme style of vitamin B to vitamin B6 intake and further increase the immunologic response of critically ill patients. Deficiency in B‐complex vitamins leads to maximum susceptibility to infections and immune diseases. Vitamin B6 deficiency leads to various impairments in immunity, such as lymphoid atrophy and reduced numbers of lymphocytes vitamin B9 deficiency diminishes the activity of NK and CD8+ T cells, which is expounded with the reduced resistance power to combat infections. The vitamin B complex is important for the metabolism of nucleic acids, amino acids, and lipids, whereas Vitamin B9 (folate or folic acid) is crucial for super molecule and protein synthesis. B‐complex vitamins help improve immunity by assembling antibodies and assisting in the communication between chemokines and cytokines. Similarly, Vitamin B5 notably prevents the expansion of Mycobacterium by maintaining the innate and adaptive immune capacity. A study showed that HIV‐infected patients who were laid low with Vitamin B6 deficiency exhibited reduced lymphocyte mutagens reactive and reduced natural CD8 T‐cell cytotoxicity compared to HIV‐positive persons who were not Vitamin B_6_ deficient. Lack of vitamin B lowers T‐cell function, antibody production, and lymphocyte expansion and proliferation. Vegan individuals are at maximum risk of developing Vitamin B12 needs malignant anemia, and of itself people rely exclusively on vegetarian diets from plant sources and abstain completely from animal products such as eggs, milk, and other dairy products, resulting in lower serum levels of vitamin B12, development of specific characteristics such as paresthesia and sore tongue, and signs of degeneration of the long tracts of the spinal cord. Although the occurrence of direct affective antiviral (DAA) therapy has remarkably maintained the cure response to hepatitis HCV infection, cure with pegylated interferon together with ribavirins is the quality of care (SOC) for the treatment of chronic HCV infections in the world with mixed medical facilities. Considering the barrier effect of B‐complex vitamins on HCV multiplication, cyanocobalamin supplementation has been found to be very effective in the treatment of patients with chronic HCV infection (Mokhtare et al., 2019). Therefore, we can conclude that regularly supplementing diets with vitamins in adequate amounts can enhance the body's natural defense mechanism against harmful toxic and viral agents by boosting the immune response (Aslam et al., 2017).

### 3.3 Role of vitamin C

**FIGURE 3:**
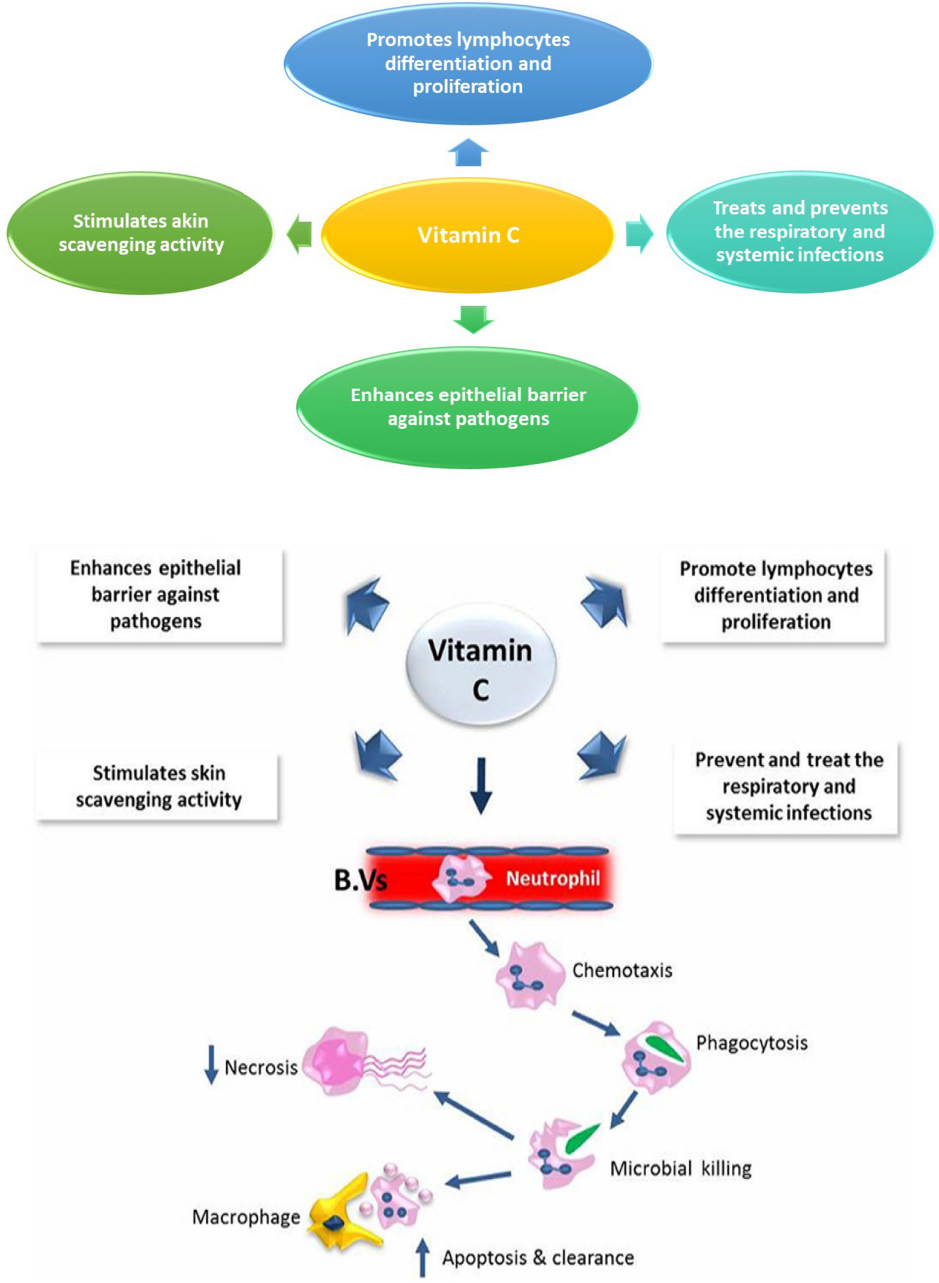
Role of vitamins C in the immune defense.

Vitamin C plays an important role in buildup immunity by supporting epithelial inhibition function against various pathogens, promoting atom scavenging activities, acting as the strongest antioxidant, protecting the body against environmental oxidative pressure, and accumulating within phagocytic cells, such as neutrophils, enhancing phagocytosis, chemotaxis, and preparation of reactive oxygen species, thereby destroying microbes, as shown in Figure 3 (Carr & Maggini, 2017). Ascorbate provides protection against communicable diseases together with cancer and other chronic degenerative diseases, aside from antioxidant and immune‐boosting activities, and acts as a safeguard against viral infections because it has the power to inactivate a good range of viruses and suppress viral replication and expression in infected cells. It has been reported that the requirement for antioxidant is notably higher in patients with HIV infection, as plasma ascorbate concentration is lower than plasma tocopherol concentrations.

Vitamin C is considered to be a potent antioxidant because it may be a free‐radical scavenger because it can participate in an atom and create a comparatively stable ascorbyl atom (i.e., L‐ascorbate anion) that may search superoxide and peroxide acid, peroxyl radicals, and oxidant air pollutants and is extremely effective against singlet oxygen, which can damage DNA, proteins, or membrane structures. Vitamin C is highly required during wound healing, as vitamin C could be a cofactor for collagen combination and a strong antioxidant, and is quickly utilized post wounding (Mohammed et al., 2016). Vitamin C deficiency delays wound healing as the formation of collagen is affected, whereas the quick healing of wounds requires the preparation of hard animal cells on the scar. Studies have indicated that antioxidant can alter organic phenomenon profiles within dermal fibroblasts, promoting fibroblast proliferation and migration for cell remodeling and wound curing. Vitamin C deficiency is one of the prominent reasons for increased susceptibility to tract infections, especially pneumonia (Hemilä, 2017). Vitamin C plays a key role in the preparation of the antiviral immunity reaction during the first phase of virus contamination through the assembly of the type I interferon, which upregulates cytotoxic and Natural T‐cell T lymphocytes, and may also be utilized as an efficient intermediate for inactivating both DNA and RNA viruses, reducing viral infectivity. According to a previous study, it acts as an antioxidant by protecting the host cells from viral‐induced radical attack. To prevent virally induced problems, the redox potential of vitamin C shields host cells from reactive oxygen species preparation from the immune‐associated respiratory burst and inflammatory response to viral infections. A high dose of vitamin C has been reported to be potent against viral infections, such as the simple cold rhinovirus Chikungunya (Gonzalez et al., 2014; Marcial‐Vega et al., 2015) avian virus H1N1, Zika (Gonzalez et al., 2016) and influenza (Zarubaev et al., 2017). Research on guinea pigs and other animals has indicated that water‐soluble vitamins modify sensitivity to several viral and bacterial infections, including pneumococcal infections.

### 3.4 Vitamin D

Vitamin D is the most potent vitamin known for strengthening bones, has rich antioxidant properties, and is manufactured in the skin from 7‐dehydrocholesterol upon exposure to sunlight. Apart from the mobilization of bone calcium and phosphorus, mineralization and formation of new bone, and participation in muscle formation. The vitamin D plays a main role in the body as well (Khan et al., 2022). Cod liver oil is a superb origin fat‐soluble vitamin that has been used for the treatment of tuberculosis and for increasing protection from infections. Vitamin D deficiency interferes with T‐cell‐mediated immunity, and immune responses mediated by T cells are often controlled by the large supplementation of calcitriol, that is, 1,25 dihydroxy‐cholecalciferol. D inhibits B and T lymphocyte (lymphocyte) proliferation and blocks lymph cell differentiation together with immunoglobulin secretion. Vitamin D inhibits monocyte preparation of inflammatory cytokines such as IL‐6, IL‐1, IL‐12, IL‐1, and TNF‐α and suppresses dendritic cell (DC) differentiation along with the maturation and storage of an imperfect phenotype as reported by a reduced effect of co‐stimulatory molecules, MHC class II molecules and IL12. It has been emphasized that Vitamin D modulates cytokine profiles in animal models of autoimmune disorders through fixed excessive production of cytokines pro inflammatory resulting in suppression of inflammation, also playing a critical role in the immune defense of the respiratory body by inactivating viral pathogens directly by the antimicrobial peptides and increased engagement of phagocytes (Duque & Descoteaux, 2014). In one study, it was stressed on the theory that fat‐soluble vitamins play a potent role in the prevention of viral infection, emphasizing the fact that there is a major relationship between the standard of Vitamin D and, therefore, the number of viral cases, especially the mortality due to this infection. In addition, it was found that the most highly affected group of the population with viral infection was elderly individuals who exhibited vitamin D deficiency (Ilie et al., 2020). One study stressed that vitamin D can regulate the macrophage effect, preventing them from delivering too many inflammatory chemokines and cytokines (Martens et al., 2020). Some cross‐sectional studies have supported the speculation that insufficiency or deficiency of Vitamin D could also be one of the most important risk factors for the progression of HIV, indicating a positive effect between CD4+ cell counts and 1,25(OH)2D. Therefore, adequate consumption of food rich in D has proven to be highly effective in the storage and treatment of viral (Yin & Agrawal, 2014).

#### 3.4.1 Mechanisms of vitamin D immune modulation

**FIGURE 4:**
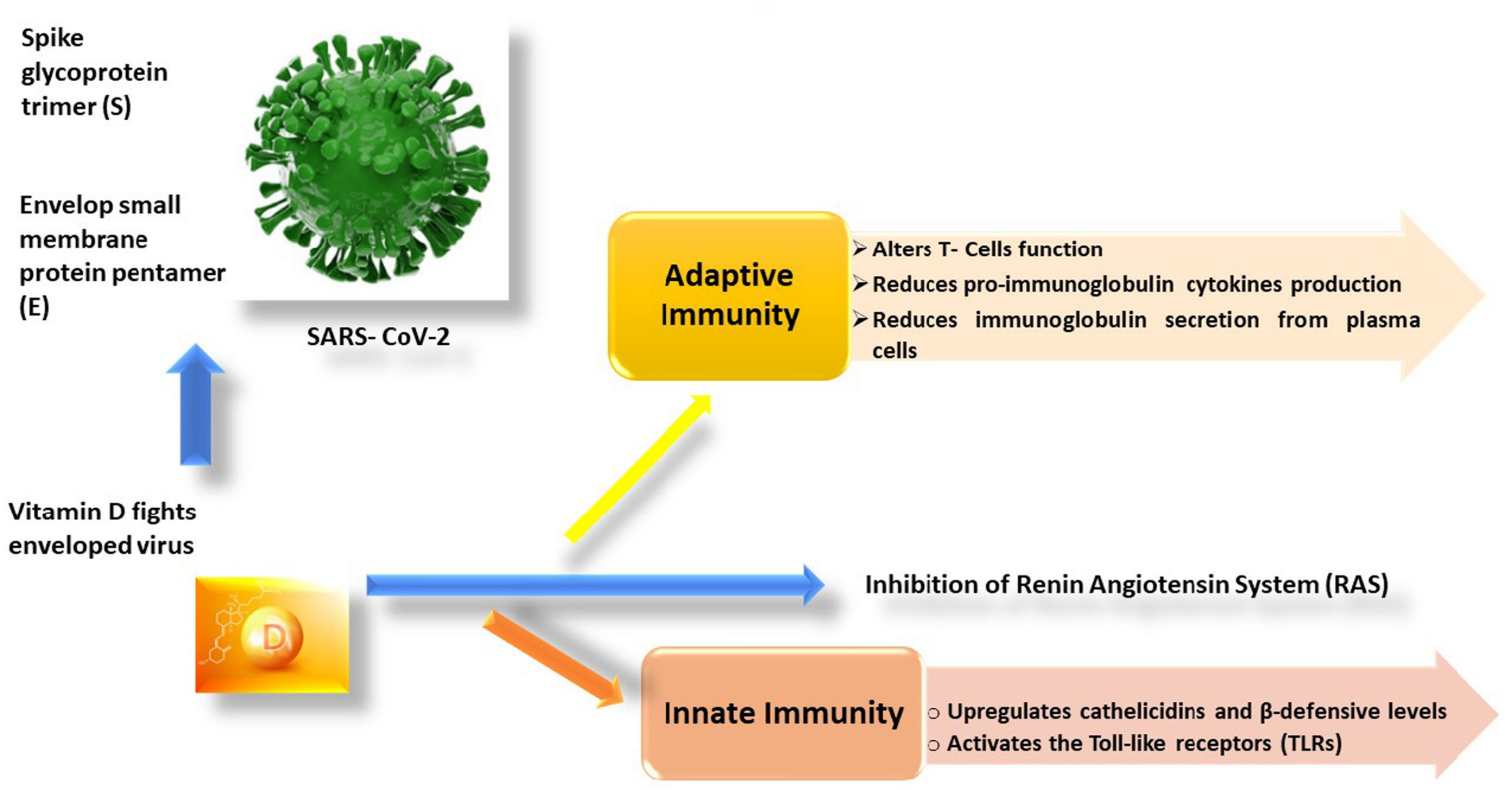
Impact of vitamin D on adaptive and innate immune effect in COVID‐19 patients.

Compound 1,25(OH)_2_VD3 has an essential impact on immune cells, such as dendritic cells (DCs), macrophages, B cells, and T cells. In macrophages and monocytes, 1,25(OH)_2_VD3 induces an increase in monocytes and cathelicidin, thus inducing innate immune reactions to some bacteria. 1,25(OH)_2_VD3 decreases IL‐12 production by DCs, IL‐17, and DC maturation, weakens the cytotoxic activity, and is liable for the proliferation of CD8+ T cells and CD4+, thus inhibiting the proliferation of B‐cell and production of immunoglobulins. The impact of vitamin D on the adaptive and innate immune effect in COVID‐19 patients was illustrated in Figure 4.

### 3.5 Vitamin E

It is a peroxyl radical scavenger that prevents polyunsaturated fatty acid lipoproteins and membranes. Vitamin E, also known as tocopherol, is primarily obtained from corn, wheat germ, nuts, olives, seeds, spinach, paragus, and other green leafy vegetables. Its excellent sources also include groundnut, soy, cotton seed, and safflower oils. As reported by a study, out of the four tocotrienols and four tocopherols (designated as β‐, α‐, γ‐, and δ) got in food, only α‐tocopherols fulfills the necessities of antioxidant in human being. It is believed that α‐tocopherols in the availability of water‐soluble antioxidants such as Vitamin C may be highly effective in food materials. Vitamin E plays a key role as a robust antioxidant when employed in combination with selenium, thereby protecting against cardiovascular diseases, especially atherosclerotic lesions, by decreasing the susceptibility to LDL oxidation, atom generation, and membrane defects, as reported in a study on guinea pigs. Vitamin E deficiency diminishes the power of the body's immunity to combat infectious microbes and mount an antibody response to antigens, and its deficiency ends up in the onset of the disease called ataxia (Jiang, 2014). A study has supported the idea that vitamin E increases T‐cell disparity through the increased TEC function within the thymus related to the increasing binding power of TEC to immature T‐cells alongside the growing effect of the adhesion molecule ICAM‐1, suggesting that fat‐soluble vitamins are powerful nutrients needed for promoting health in elderly people by improving cellular immunity that decreases with aging. An investigation in rats suggested that Vitamin E plays a very important role in T lymphocyte differentiation in the thymus, related to the antioxidant properties of vitamin E. Research conducted on HIV patients indicated that E can curb the action of the transcription elements NF‐kappa AP‐1 and B, hindering the effect of CD95 ligand protecting T lymphocyte activation‐induced necrobiosis from CD95‐mediated apoptosis; AICD could be a major explanation for lymphocyte depletion in AIDS; vitamin E is an antioxidant that is thought for its immune regulatory properties, thus boosting host protection against adverse effects of bacterial infection (Bou Ghanem et al., 2017). Increased consumption of antioxidant in the diet may result in mitogens refreshing growing T‐lymphocyte differentiation, increasing cytotoxic cell activities, and increasing the effect of macrophages against external agents, thus protecting the body against infections. Vitamin E blocks the activation of the enzyme protein kinase PKC, which reduces nitric oxide, and platelets show decreased superoxide accumulation within neutrophils and macrophage cells. As suggested by Mileva et al., Vitamin E is often used as part of multitask influenza treatment owing to its synergistic antiviral effects when used in combination with oseltamivir. Newcastle disease virus (NDV) is a highly devastating disease in chickens, which has been reported to be the cause of oxidative pressure and histopathological changes within the jejunum and duodenum of chickens, and may be partially or fully improved with the nutrition of Vitamin E (Rehman et al., 2018). Deficiency of antioxidant increases pathology in the hearts of mice contaminated with a myocarditic coxsackievirus B3 and exhibits enhanced virulence, yet stressing on the concept of increased requirements of Vitamin E. Vitamins are the most important nutrients for enhancing immunity by having positive effects on the vital organs of the human body. Vitamin D_3_ (VD_3_), the active component of vitamin D, is prepared from 7‐dehydrocholesterolin under ultraviolet B radiation of sunlight. It is mostly consumed through diet or supplements. VD_3_ is converted to 25‐dihydroxyl vitamin D_3_ 25(OH) VD_3_ in the liver, which is then metabolize to 1,25(OH)_2_VD_3_ as the highly active metabolite of VD_3_ in the kidneys. VD_3_ is also metabolized by cells of the immune system, thereby exerting positive effects on the body. 1,25(OH)_2_VD_3_ accumulates in lymphocytes containing a high amount of VD_3_ enhancing its specific action and minimizing the negative effects of the system and diseases (Bikle, 2014).

### 3.6 Fatty acids

Omega‐3 polyunsaturated fatty acid analysis has proven that omega‐3 polyunsaturated fatty acids are the most effective antioxidant among other nutrients in preventing cardiovascular disorders (Jain et al., 2015), within the treatment of depression and should enhance local inflammatory responses at wound sites (Kiecolt‐Glaser et al., 2014). Omega‐6 and omega‐3 derived metabolite are the most effective immunoregulatory components required for the proper functioning of significant organs in the body. Research indicates that Omega‐3 fatty acids have the ability to modulate cellular membrane features, such as membrane fluidity and intricate assembly in lipid rafts, and have shown immunomodulatory effects on various cells, such as Neutrophils, Eosinophils, Basophils, Dendritic cells, and T cells that conform to the immune system (Gutiérrez et al., 2019). Another study supported the characteristics of polyunsaturated fatty acids (PUFAs) withomega‐3 (DHA), arachidonic acid (AA), and omega‐3 (EPA) to exert anti‐HCV activities utilizing an HCV (hepatitis C virus) sub‐genomic RNA duplication system. Anderson et al. reported that (*n*‐3) PUFA affects the resistance to host infectious disease because it has the power to alter cytokine production and diminish eicosanoid biosynthesis. A study conducted on mice indicated that omega‐3 PUFA obtain a lipid mediator protecting PD1, which remarkably decreases influenza virus replication and protects against deadly influenza infection. Therefore, with studies showing immune‐boosting properties and protecting the body against the detrimental effects of viruses, it may be considered an efficient antivirulent that may be employed in the treatment of the present pandemic virus.

### 3.7 Minerals

#### 3.7.1 Selenium

Selenium may be a micronutrient, and the sole well‐defined metabolic action of selenium in humans is peroxidase, which plays a very important role in removing oxide and organic hydroperoxides, which, together with fat‐soluble vitamins and enzymes, form part of the antioxidant arms (Kieliszek, 2019). Among the trace elements, selenium is known for its antioxidant and redox state regulation and protection against diseases such as diabetes and some types of cancer, proving its importance as an anticarcinogenic micronutrient; however, these functions have proven to be highly beneficial for their anticarcinogenic and antioxidant properties in combating several diseases such as diabetes and several other types of cancer (Roman et al., 2014). Selenium is incredibly important for the central nervous system and the male genital parts, the parts for the correct functioning of hormones, for the muscle regulation and circulatory system, and enhancing system when consumed in adequate amounts in the diet on a regular basis. Deficiency of Selenium leads to Keshan disease, which is associated with cardiovascular disease, coronary failure, enlargement of the heart, hypertension, arthritis, infections, and Kashin–Beck disease, which causes deformities in bones, stunted growth, joint stiffness, and chronic arthritis with restriction in movements. Besides these disorders, other problems due to its deficiency still arise, such as increased infertility in men, glandular carcinoma, and neural diseases (Shreenath et al., 2021). According to a previous study, data derived from tissue culture and animal models were compared with the consequences of Se levels on inflammatory or immune‐related diseases, including autoimmunity, antiviral immunity, sepsis, chronic inflammatory disorders, and allergic asthma, which emphasized its role as an immunostimulatory agent that plays a crucial role in inflammation and immunity. Another study has indicated that utilization of Se‐fortified broccoli may change immune responses toward a level of immune challenge by increasing cytokines in human peripheral blood mononuclear cells, and the activation of human blood leukocytes has been shown to extend the reaction to selenium‐enriched foods (Bentley‐Hewitt et al., 2014). A study in mice has shown that cloned and sequenced amyocarditic coxsackie virus B3, which creates no pathology in the hearts of Se‐adequate mice, exhibited large cardiac pathology in Se‐deficient mice. Selenium is a micronutrient necessary for antioxidant defenses and for enhancing the immune system when deficient in diet causes a decrease in the number of faster progression of AIDS, CD4 T cells, and a 20% increase in the risk of death. Studies using mouse models have shown that selenium (Se)‐deficient mice are more prone to severe pneumonitis and viral infections than Se‐adequate mice, indicating an intense effect on the genome of RNA viruses as a low nutritional condition, which is liable for the inception of the latest viral strains (Guillin et al., 2019). Patients with HCV and HIV‐co‐affected coinfection displayed notably lower blood Se levels than HIV‐positive patients without attendant HCV contamination. Any deterioration of type 2 deiodinase reactions in selenium needs has a severe impact on the system through suboptimal performance and the development of killing invading pathogenic bacteria, thymic cells, and fungi, which are crucial for different aspects of the cell‐mediated immune system, including the destruction of neoplastic cells and removal of viruses. Feasible mediators of those Se activities are the GPx, although this cannot all be direct associated with safety against peroxide. Selenium has been proven to be the most effective antioxidant and antiviral; therefore, its supplementation is utilized in the treatment of the detrimental effects of viral (Avery & Hoffmann, 2018).

#### 3.7.2 Zinc

Zinc is known for its excellent antioxidant properties and plays a significant role in immunity from the barrier of the skin to gene operation within lymphocytes, which is critical for the natural growth and functioning of cell‐dependent nonspecific immunity, such as natural killer cells and neutrophils. Zinc is a component of over 300 metalloenzymes, which makes it vital for several fundamental life processes. Zinc deficiency leads to chronic renal disease, syndrome, cirrhosis of the liver, rbc disease, acrodermatitis enteropathica (AE), and other chronic debilitated diseases. Together with growth decline, skin changes, male hypogonadism, poor appetite, delayed wound healing, and mental lethargy are manifestations of chronic zinc deficiency in humans. Severe deficiencies of zinc are related to dermatological manifestations resulting in phrynoderma, diarrhea, alopecia, mental disturbances, infertility in men, and intercurrent infections (Willekens & Runnels, 2022).

Studies have shown that disease deficiency results in increased susceptibility to viral infections such as HIV or hepatitis C virus. Zinc supplementation is used as a therapeutic agent for treating viral infections such as herpes simplex virus and the common cold (Read et al., 2019). A study done on mice models has shown that mice kept on zinc‐deficient diets had lower numbers of T lymphocytes and T‐cell subsets, diminished proliferation in response to mitogens and certain antigens, and reduced interleukin‐2 preparation (IL‐2) compared to mice fed zinc‐containing diets together with decreased levels of antibody to the T‐dependent antigen DNP‐human albumin. Cell culture analysis showed that high Zn^2+^ concentration, along with the mixing of compounds stimulating cell imports of Zn^2+^, such as hinokitiol, pyrrolidinedithiocarbamate, and pyrithione, inhibited the duplication of RNA viruses, respiratory syncytial virus with influenza virus, and several picornaviruses. Therefore, Zinc is an immune enhancer that is effective in protecting against viral infections and can be employed in the prevention of viral (Karim et al., 2021).

#### 3.7.3 Iron

Iron is a vital micronutrient needed for the growth of parasites, bacteria and neoplastic cells together with strengthening immune system by proliferating and proliferation of immune cells individual lymphocytes, related to the generation of a particular response to infection. Studies have proven that Iron deficiency results in the increased susceptibility to infection in both individuals and experimental animal models and oral supplementation of iron has indicated a reduction in infectious morbidity. In vivo and in vitro studies have shown that iron chelators, such as desferrioxamine, inhibit phagocyte oxidase activity and decrease reactive oxygen intermediate‐dependent killing of bacterial infections. The increased level of iron is related to the susceptibility and response to HBV infection; therefore, the possible relationship between higher levels of iron and HBV infection progresses to carcinoma (primary cancer of liver cells) (Gupta et al., 2022).

#### 3.7.4 Magnesium

Magnesium is one of the most important micronutrients, sharing a powerful relationship in boosting the immune system in all innate and ought immunologic effects. Mg enhances immune responses as a cofactor for the production of immunoglobulins, immune system adhesion, antibody‐dependent cytolysis, binding of IgM to lymphocytes, macrophage effects on lymphocytes, adherence of T helper B cells, binding of substance P to lymphoblasts, and antigen binding to macrophage RNA. Mg deficiency accelerates thymus involution and causes early cytological and immunological modifications within the spleen, which appear before macroscopic changes in this organ, and such changes are associated with the altered immunologic response of magnesium deficient animals. Reduced magnesium levels are related to neurodegenerative diseases, including traumatic brain injury, migraine, cocaine exposure, ethanol intoxication, stroke, subarachnoid hemorrhage, and mood disorders. Research has shown that magnesium supplementation in Magnesium Transporter 1 (MAGT1)‐deficient patients restores intracellular free Mg^2+^ and NKG2D while reducing Epstein–Barr virus (EBV)‐infected cells in vivo. This demonstrates a link between NKG2D cytolytic activity and EBV antiviral immunity in humans, indicating a selected molecular function of free basal intracellular Mg^2+^ in eukaryotic cells. Research has shown that Mg could be a potent immune enhancer that is very effective in protecting the body against viral infections (Ravell et al., 2014).

### 3.8 Other compounds

Polyphenols are bioactive compounds containing an –ON group attached to the benzene ring and are generally found in vegetables and food. They are classified as flavonoids, tannins, phenolic acids, stilbenes, and lignans, which are further divided into isoflavones, flavones, flavonols, flavonones, flavononols are best known for his or her anti‐inflammatory, antioxidant, and anticancer properties. An investigation of mouse models has shown that oral supplementation of polyphenol‐rich extracts from mature date fruit, Particularly in Peyer's spleen and patches, chlorogenic acid, caffeic acid, pelargonin, and ferulic acid from palm trees enhanced the number of immunocompetent cells, such as natural killer (NK), T helper 1 (Th1), dendritic cells (DCs), and macrophages. The most common flavonols are quercetin, myricetin, and kaempferol. Quercetin is the most important flavonol present in onions, apples, kale, and tea. With regard to anti‐inflammatory properties, the polyphenols curcumin, resveratrol, fisetin, apigenin, rutin, and quercetin were analyzed for their immunomodulatory characteristics and antiviral effects, where only fisetin and quercetin were shown to downregulate the preparation of proinflammatory cytokines by dengue virus infection‐2 and DENV‐3 infection enhanced by antibodies inhibiting IL‐6 and TNF‐α secretion (Jasso‐Miranda et al., 2019). Among the bioactive infections, polyphenols play a very important role as anticarcinogenic, antiviral, antioxidant, anti‐inflammatory, antimicrobial, and inhibit replication of the HIV virus, thus proving to be effective against viral infections (Landivar et al., 2021). The potential foods used in the intervention are presented in Table 1.

**TABLE 1:** Potential foods used for intervention.

Potential foods	Used against disease	References
Vitamin C rich foods such as oranges, lemons, strawberries, blueberries, tomatoes, broccoli, leafy greens like kale, and bell peppers	Vitamin C might play a role: cancer (including prevention and treatment), cardiovascular disease, age‐related macular degeneration (AMD) and the common cold	Li et al. (2007), Carr et al. (1999)
Omega‐3‐fatty acid such as fish, avocado, nuts, olive oil, soy, canola, sunflower and corn oils sunflower seeds, almonds, walnuts, and pecans	Long chain omega‐3 fatty acid may help to boost the immune system by enhancing the functioning of immune cells	Gutiérrez et al. (2019)
Garlic and Onions (quercetin)	Influenza A viruses (IAVs), highly pathogenic avian influenza A (H5N1) virus	Gorinstein et al. (2008), Cinatl et al. (2003)
Yogurt	Rich in probiotics Fight off viruses	Lee et al., 2017
Herbal tea/Kadha made from Tulsi (Basil), Dalchini (Cinnamon), Kalimirch (Black pepper), Dry Ginger and Munakka (Raisin)	Helpful in flu and different allergic problems	Poswal et al. (2019), Chacko et al. (2010)
Golden Milk‐ Half tea spoon Haldi (turmeric) powder in 150 mL hot milk	Milk with turmeric powder mixed in it is considered to be an ideal treatment for cough	Jagetia et al. (2007)
Foods rich in resveratrol such as peanuts, pistachios, grapes, red, white wine, blueberries, cranberries, strawberries, and even cocoa and dark chocolate	Fight fungal infection, ultraviolet radiation, stress, and injury. They also protect the body against viral attacks	Malaguarnera et al. (2019)
Star anise (spice)	It is used in treating influenza virus. It is super powerful as an antiviral	Jayant et al. (2020)
Ginseng, Astragalus, Licorice, Echinacea, Peppermint, fennel, oregano	Fighting viral infections like herpes and influenza, hepatitis B, norovirus, and coxsackieviruses	Im et al. (2016), Cinatl et al. (2003), Shi et al. (2014), Khanna et al. (2021)

## 4 CONCLUSION

Due to the emergence of viruses which created the pandemic COVID‐19 throughout the world and the unavailability of a particular curing and medication, the emphasis is now on strengthening the immunity by consuming vitamins, minerals, and other bioactive compounds. Many research studies have shown that apart from boosting the immune system, nutrients such as Vitamin A and B‐complex vitamins, Vitamin D, C, E, and minerals such as selenium, zinc, magnesium, and iron, along with bioactive compounds such as polyphenols, are not just free radical scavengers but can be used in the avoidance and curing of viral infections. Studies have shown that they are effective against viral infections, such as influenza virus, SARS, MERS, HIV virus, Hepatitis B virus, several picornaviruses, and respiratory syncytial virus. Therefore, to prevent the deadly effect of coronavirus, nutrient‐rich foods must be consumed regularly in abundance, and the components of the vitamins, minerals, and bioactive compounds can be extracted to produce a drug that can serve as a potent therapeutic agent for combating the virus.

## AUTHOR CONTRIBUTIONS

**Sonal Prasad:** Conceptualization (equal); data curation (equal); investigation (equal); methodology (equal); resources (equal); validation (equal); writing – original draft (equal). **Vinay Kumar Pandey:** Conceptualization (equal). **Kunal Singh:** Conceptualization (equal); investigation (equal); methodology (equal); resources (equal); writing – original draft (equal). **Rafeeya Shams:** Visualization (equal). **Rahul Singh:** Conceptualization (equal); supervision (equal); writing – original draft (equal); writing – review and editing (equal). **Gulden Goksen:** Conceptualization (equal); funding acquisition (equal); investigation (equal); resources (equal); supervision (equal); visualization (equal); writing – review and editing (equal).

## Data Availability

No data has been generated or produced during the preparation of this manuscript.
